# Differential Gene Analysis of Trastuzumab in Breast Cancer Based on Network Pharmacology and Medical Images

**DOI:** 10.3389/fphys.2022.942049

**Published:** 2022-07-08

**Authors:** Yuan Lu, Juan Bi, Fei Li, Gang Wang, Junjie Zhu, Jiqing Jin, Yueyun Liu

**Affiliations:** ^1^ Shanghai Eighth People’s Hospital, Shanghai, China; ^2^ Department of Pharmacy, First Affiliated Hospital, Naval Medical University, Shanghai, China

**Keywords:** trastuzumab, breast cancer, network pharmacology, molecular docking, bioinformatics, medical images

## Abstract

The purpose of this study was to use network pharmacology, biomedical images and molecular docking technology in the treatment of breast cancer to investigate the feasible therapeutic targets and mechanisms of trastuzumab. In the first place, we applied pubchem swisstarget (http://www.swisstargetprediction.ch/), (https://pubchem.ncbi.nlm.nih.gov/) pharmmapper (http://lilab-ecust.cn/pharmmapper/), and the batman-tcm (http://bionet.ncpsb.org.cn/batman-tcm/) database to collect the trastuzumab targets. Then, in NCBI-GEO, breast cancer target genes were chosen (https://www.ncbi.nlm.nih.gov/geo/). The intersection regions of drug and disease target genes were used to draw a Venn diagram. Through Cytoscape 3.7.2 software, and the STRING database, we then formed a protein-protein interaction (PPI) network. Besides, we concluded KEGG pathway analysis and Geen Ontology analysis by using ClueGO in Cytospace. Finally, the top 5 target proteins in the PPI network to dock with trastuzumab were selected. After screening trastuzumab and breast cancer in databases separately, we got 521 target genes of the drug and 1,464 target genes of breast cancer. The number of overlapping genes was 54. PPI network core genes include GAPDH, MMP9, CCNA2, RRM2, CHEK1, etc. GO analysis indicated that trastuzumab treats breast cancer through abundant biological processes, especially positive regulation of phospholipase activity, linoleic acid metabolic process, and negative regulation of endothelial cell proliferation. The molecular function is NADP binding and the cellular component is tertiary granule lumen. The results of KEGG enrichment analysis exhibited four pathways related to the formation and cure of breast cancer, containing Drug metabolism, Glutathione metabolism, Pyrimidine metabolism and PPAR signaling pathway. Molecular docking showed that trastuzumab has good binding abilities with five core target proteins (GAPDH, MMP9, CCNA2, RRM2, CHEK1). This study, through network pharmacology and molecular docking, provides new pieces of evidence and ideas to understand how trastuzumab treats breast cancer at the gene level.

## Introduction

Globally, as the most commonly diagnosed women, malignant disease breast cancer (BC) represents 24% of new cancer cases, and its incidence rate is still on the rise (Heer et al., 2020). It is the second main reason for death in women with cancer, making up 15% of cancer death in 2018 (Liang et al., 2020). The mortality rate has not increased with the morbidity rate due to the advanced clinical treatment and prevention, but the numbers cannot be underestimated (Schmidt et al., 2017). The risk factors include age at the important period of women, race, exogenous hormones, BMI, alcohol consumption and radiation exposure and so on (Mehraj et al., 2021). The treatment options and prognosis of breast cancer mainly depend on tumor-node-metastasis staging. And it is also influenced by other vital factors such as histology, the state of hormone receptor, overexpression of ERBB2, coexisting disease, and menopausal condition (Wang et al., 2021).

Overexpression of the HER2 (Human egfr-related 2) transmembrane receptor, indenfied by immunohistochemistry or enhancement of the HER2/neu gene located on chromosome 17, is the trait of breast cancer or HER2-positive. (Bartsch, 2021). The type I (EGFR-related) involved in the biological behavior of breast cancer cells is HER2 growth factor receptor, which gene is overexpressed in about 15–20% of breast cancers and represents a worse prognosis (Perez et al., 2013). As a humanized monoclonal antibody that combines with HER2 particularly, Trastuzumab (Herceptin, Roche), was proven for patients with metastatic breast cancer overexpressing HER2 (Burstein, 2020) and should be administered with chemotherapy (Chan et al., 2020). Trastuzumab is relatively safe and well-tolerated. The most common adverse events are fever and chill related to infusion, and other frequent adverse effects include nausea, vomiting, infections, neutropenia and so on. For the early and advanced HER2-positive breast cancer treatment, due to the efficacy and accepted adverse reactions, trastuzumab has been the first choice (Maximiano et al., 2016).

Biomedical image is a new research method, which focuses on computer, mathematics, biomedical engineering and other subjects. Biomedical images can visualize numbers and codes, and explore the medical rules therein, so as to apply them to clinical practice. By using network pharmacology and biomedical images, this study will provide a new angle to explain the mechanism which trastuzumab treats breast cancer clearly.

## Methods

### Filtering of Target Proteins of Trastuzumab

In pubchem (https://pubchem.ncbi.nlm.nih.gov/), we searched for the keyword Trastuzumab, and the structural formula was downloaded by us. Data were collected from three websites, pharmmapper (http://lilab-ecust/pharmmapper/), swisstarget (http://www.swisstargetprediction.ch/), and BATMAN-TCM (http:/bionet.ncpsb. org/BATMAN-TCM/) databases according to the structural formula. The swisstarget database filters data using Probability * >0; the batman-tcm database filters data using Score cutoff > = 5; the pharmmapper database has no data filter. The data downloaded from the pharmmapper database is transformed into the corresponding gene using the Uniprot database (http: www.Uniprot.org/).

### Screening of Gene Targets for Breast Cancer

Search the keyword breast cancer in NCBI-GEO () and” title = "https://www.ncbi.nlm.nih.gov/geo/)and">https://www.ncbi.nlm.nih.gov/geo/)and select gene expression studies containing 41 breast cancer and 24 normal breasts.

### Venn Diagram

Take the intersection of the target genes of trastuzumab and therapeutic targets for breast cancer and draw Venn diagram using Venny2.1 (https://bioinfogp.cnb.csic.es/tools/venny/). 521 drug targets, 1,464 changed disease genes, and 54 intersection targets were obtained. All the duplications were deleted.

### Establishment of Protein-Protein Interaction (PPI) Network

PPI data were obtained from the STRING database, with a 0.4 min interaction score. Also, the delete outliers were set. Construct PPI network using Cytoscape3.7.2 software. Use R software to import PPI data and get the number of connected nodes of core genes.

### Enrichment Analysis

Through ClueGO in Cytoscape, common target Gene Ontology (GO) and Kyoto Encyclopedia of Genes and Genomes (KEGG) enrichment analyses were obtained. Molecular function (MF), biological process (BP), and cellular component (CC) were included in the GO enrichment analysis (Bartsch, 2021) The results are presented in bar charts, pie charts and network charts.

### Molecular Docking

For further checking the reliability of the prediction results, the molecular docking verification was carried out with the most active components and the main related targets. First, Download the active ingredient’s 2D structure from PubChem (https://pubchem.ncbi.nlm.nih.gov/) and convert it to 3D using ChemOffice software. Then, protein receptors were collected from the PDB database (http://www.rcsb.org/), and water molecules were also removed. Also, small molecular ligands using PyMOL software. Use AutoDockTools-1.5.6 to hydrogenate the protein and confirm the active pockets of the proteins. Finally, molecular docking was done by vina software.

## Results

### Select the Target Genes of Drug and Disease

**FIGURE 1:**
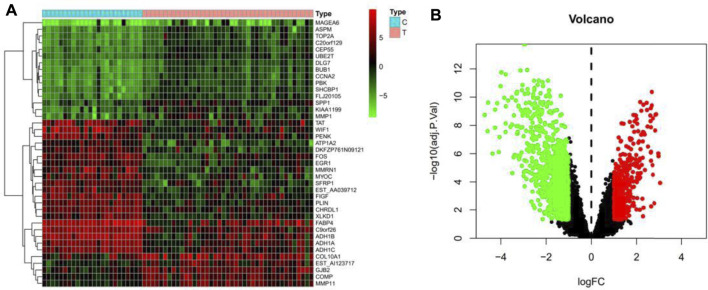
**(A)**The heatmap of breast cancer target genes; **(B)**The volcanic map of breast cancer target genes. Green represents down-regulated genes; black represents no difference in genes, red represents up-regulated genes.

From swisstarget (http://www.swisstargetprediction.ch/, pharmmapper (http://lilab-ecust.cn/pharmmapper/), batman-tcm (http://bionet.ncpsb.org.cn/batman-tcm/) database, trastuzumab was searched. After removing duplicate targets, a total of 521 genes were gained. The result is shown in Table 1. Breast cancer was searched in NCBI-GEO (https://www.ncbi.nlm.nih.gov/geo/) database and obtained the information of all genes, which were presented by heatmap in Figure 1A and volcanic map in Figure 1B.

**TABLE 1:** Overlapping targets of trastuzumab and breast cancer.

TCN1 TGFBR2 CHEK1 PLEKHA4 NR3C1 NPY2R
UHRF1 ICAM2 LYZ STAT1 RND3 P2RY12
HELLS ANG PCK1 NMNAT3 ADAM33 POLQ
ACVRL1 CTSB CAT F10 TAP1 ESCO2
ACSL1 AMY2A KIT SDS MAOA LAMA2
ABCB1 CFB TK1 GSTA1 TUBB3 DPYSL2
DPP4 CCNA2 CTSF GSR TAC1 CX3CR1
GAPDH UCK2 MMP9 KIF11 NGFR RRM2
CD38 GALE MMP1 GSTM2 ZBTB4 POLE2



**FIGURE 2:**
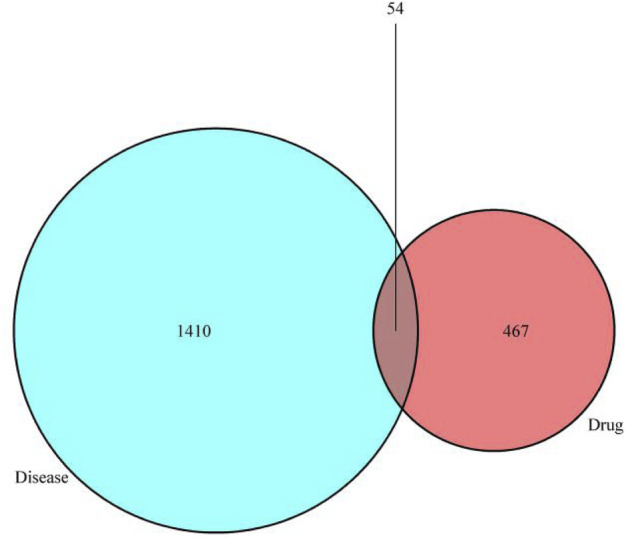
Venn diagram: The 54 overlapping genes between trastuzumab and breast cancer.

As is shown in biomedical images, 54 genes were obtained by eliminating the duplication of drug targets and disease targets, which are the interactive targets pf trastuzumab; those genes are shown in Table 1. Venny2.1 (https://bioinfogp.cnb.csic.es/tools/venny/) was used to generate Venn diagram, which is shown in Figure 2.

### Establishment and Analysis of Target Proteins PPI Network

**FIGURE 3:**
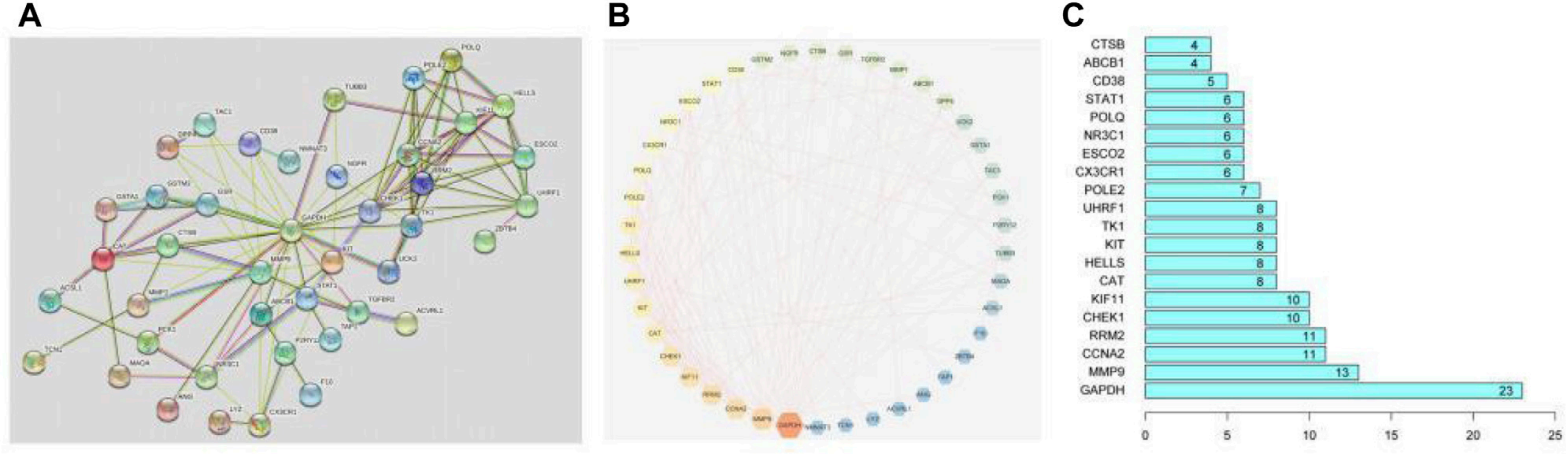
PPI network and its analysis.

The interacting proteins form a protein-protein interaction network (PPI). It plays a role in gene expression regulation, biological signal transmission, cell cycle regulation, energy metabolism, and other life phrases. To explore the interaction between these overlapping genes, the detail of them was uploaded to the STRING database and formed a PPI network, as shown in Figure 3A. Set the minimum score threshold to >0.4. The interaction between these proteins is denoted by edges. In a PPI network, the more lines, the higher the relevance and the higher the target rank. Next, we imported the results to Cytoscape 3.7.2 software to select 42 core genes, as shown in Figure 3B. The brighter and larger the color in the figure, the higher the degree value, and the more important the corresponding node in the network is. And the 20 core genes in PPI network is shown in the bar plot in Figure 3C. As a result, the PPI network describes a complicated association between these genes.

1) PPI network of 42 core targets. The brighter and larger the graph is, the higher the degree value is, and the more important the corresponding node in the network is; 2)The protein-protein interaction (PPI) network of 54 overlapping genes. The higher the number of lines, the higher the correlation and the higher the target rank; 3) Top 20 genes in the bar plotinPPI network.

### Gene Ontology (GO) Enrichment Analysis

**FIGURE 4:**
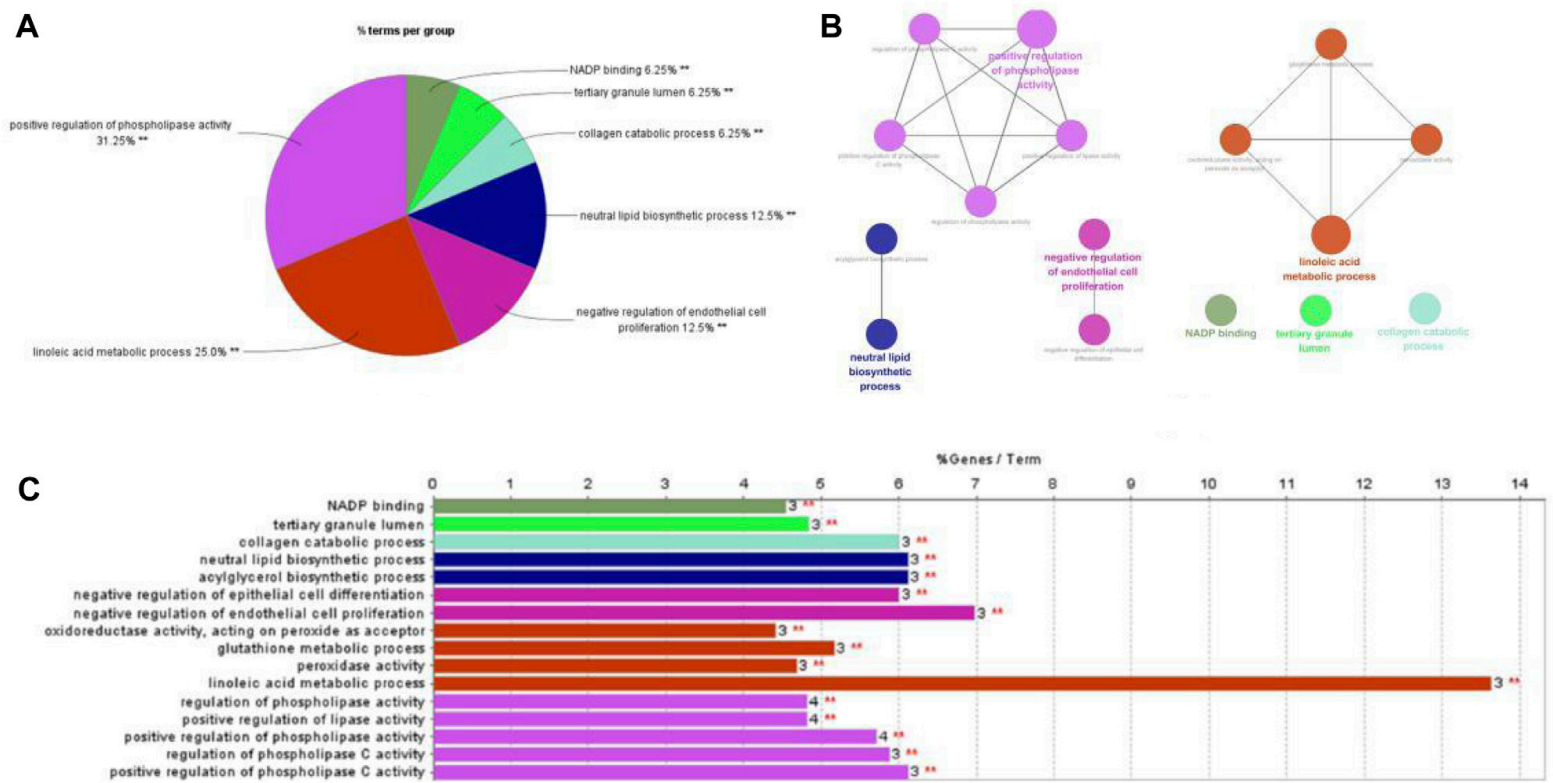
GO analysis in trastuzumab treated with breast cancer.

As a widely used Ontology in bioinformatics, 3 biology areas are involved by Gene Ontology: molecular function, cellular component, biological process. In order to figure out how these 54 genes affect breast cancer, GO enrichment analysis was carried out by ClueGO in Cytoscpace. GO enrichment analysis contains a biological process (BP), molecular function (MF), and cellular component (CC). As shown in Figure 4, the lenoic acid metabolic process and negative regulation of endothelial cell proliferation are two important process in the treating course, accounting for 25% and 12.5% separately. The results indicated that trastuzumab treats breast cancer through abundant biological processes, especially linoleic acid metabolic process (GO:0,043,651), positive phospholipase activity regulation (GO:0,010,518), and negative endothelial cell proliferation regulation (GO:0,001,937).

### KEGG Pathway Enrichment Analysis

**FIGURE 5:**
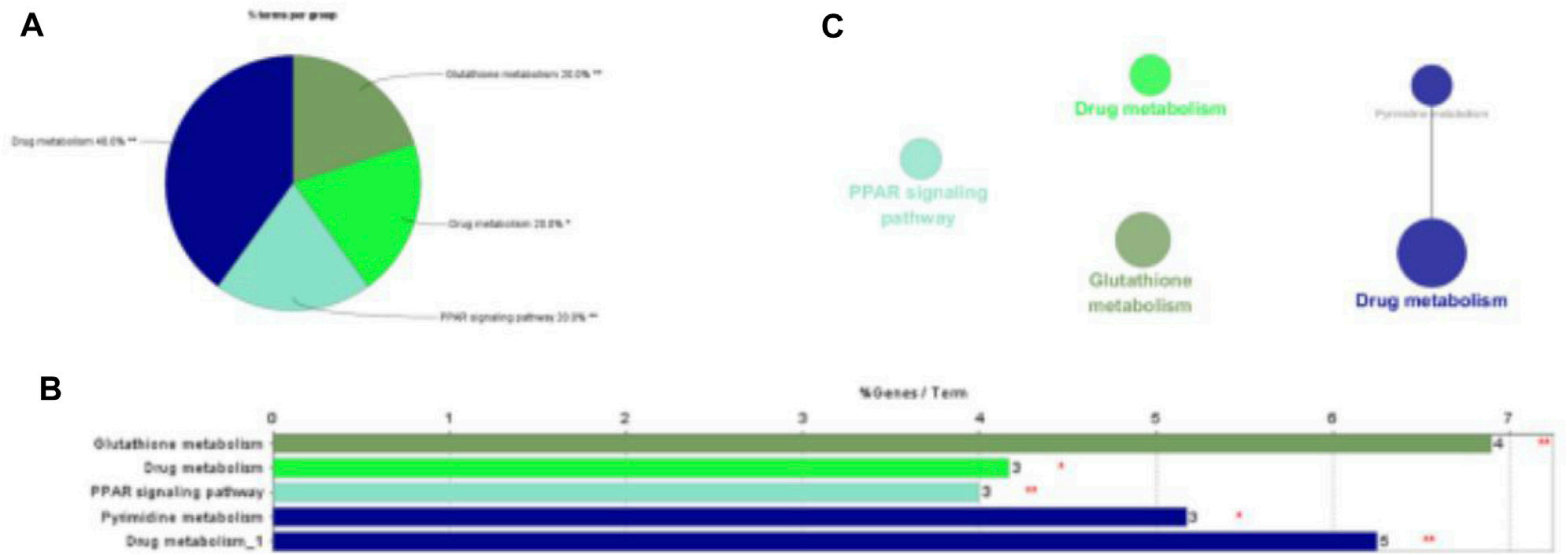
KEGG pathway enrichment analysis in trastuzumab treated with breast cancer.

Like cells, organisms, and ecosystems, a database resource that comprehends the senior roles and biological systems used from genomic and molecular degree information refer to KEGG. We uploaded 54 overlapping genes into Cytoscpace’s ClueGO to deeply investigate how trastuzumab used these target genes to influence breast cancer. The KEGG enrichment analysis results are shown in the pie chart, bar chart and network graph as shown in Figure 5. 54 overlapping genes were uploaded to ClueGO in Cytoscpace, and the results of KEGG enrichment analysis are shown in the pie chart, bar chart and network chart in Figure 5. KEGG enrichment analysis demonstrates four pathways that core genes are enriched to influence breast cancer, including Drug metabolism, Glutathione metabolism, Pyrimidine metabolism and PPAR signaling pathway.

### Molecular Docking

**FIGURE 6:**
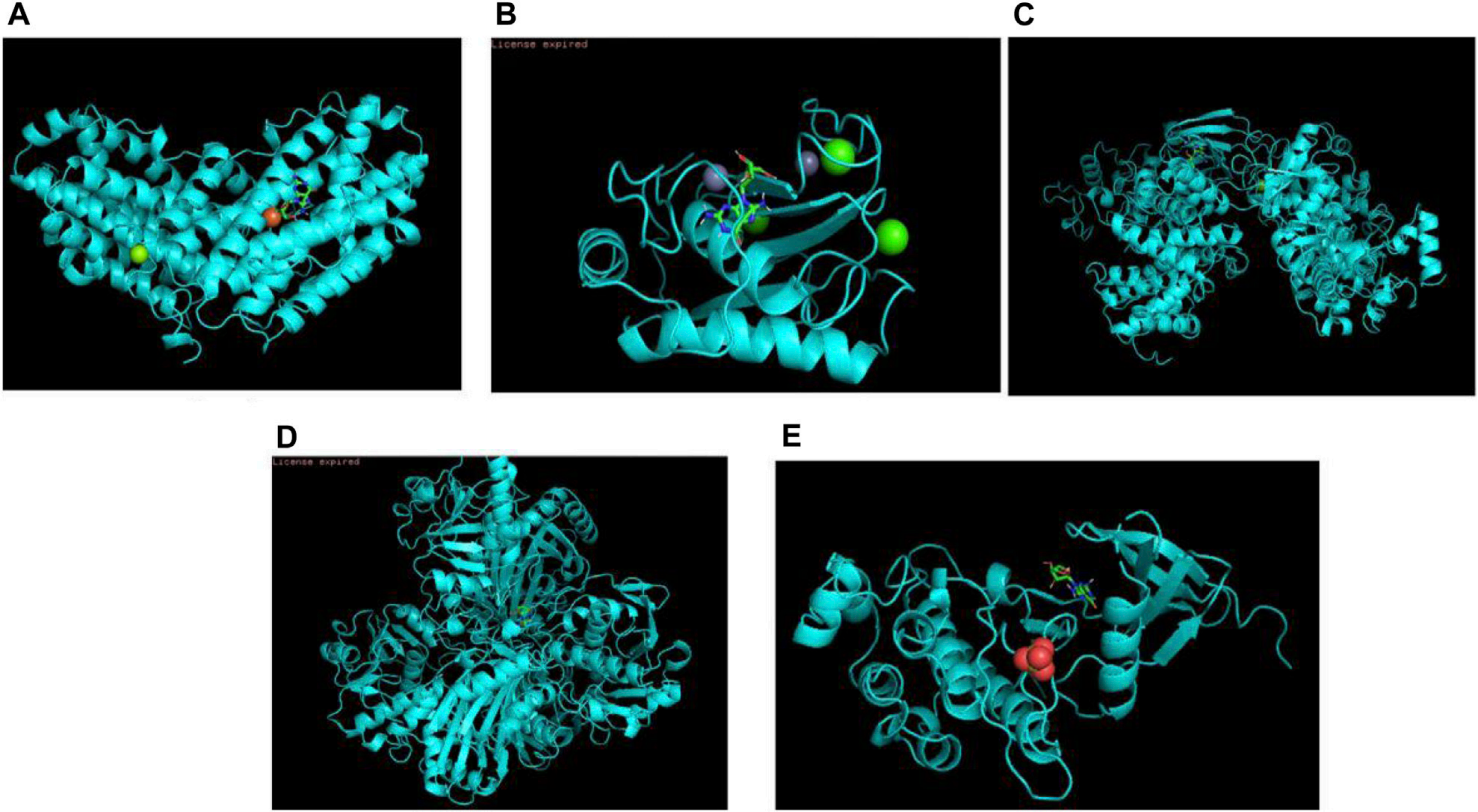
Docking patterns of trastuzumab to core targets. **(A)** Docking pattern of trastuzumab to GAPDH; **(B)** Docking pattern of trastuzumab to MMP9; **(C)** Docking pattern of trastuzumab to CCNA2; **(D)** Docking pattern of trastuzumab to RRM2; **(E)** Docking pattern of trastuzumab to CHEK1.

Molecular docking is a way to visualize information at the molecular level using biomedical image. The approach to medicine design that is based on receptor nature and interactions between drug molecules and receptors refers to a theoretical simulation approach called molecular docking. The purpose of this way is to detect intermolecular interactions (such as ligand and receptor). As is shown in biomedical images, their binding modes and affinity would be predicted as well. In computer-aided medical research field, molecular docking techniques have gained increasing importance in recent years. According to the PPI network, we selected five key target proteins GAPDH, MMP9, CCNA2, RRM2, CHEK1 and molecularly docked these proteins with trastuzumab. When the binding energy is less than 0 kcal/mol, the spontaneous binding and interaction of molecular proteins are considered, and the molecular conformation is more stable when the binding energy is lower. If the binding force is less than −5.0 kcal/mol, it means the two have a good combination. Table 2 and Figure 6 shows the result of the binding energy and docking diagrams separately. Each key protein’s binding energy to trastuzumab was less than-6.9 kcal/mol, which indicated that each protein could bind to trastuzumab well.

**TABLE 2:** Binding energy of trastuzumab with core target genes.

Target Protein	Binding Energy (Kcal/Mol)
GADPH	−8.2
MMP9	−9.1
CCNA2	−7.7
RRM2	−7.4
CHEK1	−6.9

## Discussion

Approximately 15–20% of breast cancer patients overexpress the HER2 gene, leading to poor outcomes, making it the most prevalent malignancy in women all over the world (Herceptin, Roche), has been the first-line treatment of early and advanced HER2-positive breast cancer (Fotis et al., 2018; Padrón et al., 2020). Trastuzumab is a kind of humanized anti-HER2 monoclonal antibody. Network-based technologies and biomedical images help advance the mechanism-based concept of drug discovery and strengthen the early stages of drug discovery by illuminating the basic biology of drugs and diseases (Zhang et al., 2015; Boezio et al., 2017). Network pharmacology provides the potential to explore the drug’s function in the whole physiological environment, assisting us to get a better understanding of the drug-target-disease interaction (Mueller et al., 1998; Kotta-Loizou et al., 2012).

In this research, 54 duplicated genes of trastuzumab and breast cancer were selected by using public database and biomedical images. And through the construction and analysis of the PPI network, we got several key genes such as GADPH, MMP9, CCNA2 and so on. In the images we obtained in the study, GAPDH is down-regulated in various cancer cells. Besides its role in energy metabolism, GAPDH can also interfere with the cancer cells’ destiny, for which GAPDH is able to serve as a regulator of cell death. So some studies have regarded GAPDH as an alternative therapeutic target (Early and Moon, 2021; Trayes and Cokenakes, 2021). As found by biomedical images, MMP9 is an extracellular matrix associated with stromal cell protein that promotes tumor progression and regulates the activity of cell adhesion molecule and cytokines. MMP9 is a matricellular protein causing extracellular matrix (ECM) remodelling, so it can stimulate tumour progression, and influence the function of and cell adhesion molecules. The expression of MMP9 was positive in matrix and cytoplasm of breast cancer cells, and the increase of MMP9 protein levels was related to high tumor grade (Zhang and Plitas, 2021). So trastuzumab may treat breast cancer by affecting these tumor-related genes.

As is shown in biomedical images, GO enrichment analysis presented that trastuzumab influences breast cancer mainly through linoleic acid metabolic process, positive regulation of phospholipase activity, and negative endothelial cell proliferation pathways regulation. The molecular function of trastuzumab target proteins is NADP binding. KEGG pathway enrichment analysis showed four pathways related to the development and treatment of breast cancer, including Drug metabolism, PPAR signaling pathway, Glutathione metabolism, and Pyrimidine metabolism. Intracellular transcription factors activated by specific ligands, Peroxisome proliferation-activated Receptors (ppar) are part of the nuclear hormone receptor superfamily (Zarwan et al., 2021). The growth of human breast cancer cells is inhibited by overexpression of PPAR in human breast cancer cell lines, triggering serious lipid cumulation and promoting cell phenotype changes, and leading to the increase of cell differentiation and the decrease of malignant degree (Román et al., 1007). Several PPAR agonists have been confirmed as potential tools to inhibit breast tumor growth and progression. Trastuzumab may activate PPAR signaling pathway or up-regulate the expression of PPAR to treat breast cancer. Biomedical images and molecular docking indicates that the binding energy of each key protein to trastuzumab was less than −6.9 kcal/mol, meaning that the combination between each protein and trastuzumab is tight.

However, the results of this study are limited due to the incomplete information in the database and inadequate gene expression studies. Moreover, this study has not been able to verify the possible molecular mechanism of how trastuzumab cures breast cancer *in vivo* and *in vitro* by carrying out experiments, but fresh perspectives and aspects are presented for future related experiments.

## Data Availability

The original contributions presented in the study are included in the article/Supplementary Material, further inquiries can be directed to the corresponding author.
